# The company they keep: Background similarity influences transfer of aftereffects from second- to first-order stimuli

**DOI:** 10.1016/j.visres.2013.05.008

**Published:** 2013-07-19

**Authors:** Ning Qian, Peter Dayan

**Affiliations:** a Department of Neuroscience, Columbia University, New York, NY 10032, USA; b Department of Physiology & Cellular Biophysics, Columbia University, New York, NY 10032, USA; c Gatsby Computational Neuroscience Unit, University College London, London WC1N 3AR, United Kingdom

**Keywords:** Aftereffect transfer, Contingent aftereffects, Cross-order adaptation, Subjective contours, Illusory contours, 1/*f* noise

## Abstract

A wealth of studies has found that adapting to second-order visual stimuli has little effect on the perception of first-order stimuli. This is physiologically and psychologically troubling, since many cells show similar tuning to both classes of stimuli, and since adapting to first-order stimuli leads to aftereffects that do generalize to second-order stimuli. Focusing on high-level visual stimuli, we recently proposed the novel explanation that the lack of transfer arises partially from the characteristically different backgrounds of the two stimulus classes. Here, we consider the effect of stimulus backgrounds in the far more prevalent, lower-level, case of the orientation tilt aftereffect. Using a variety of first- and second-order oriented stimuli, we show that we could increase or decrease both within- and cross-class adaptation aftereffects by increasing or decreasing the similarity of the otherwise apparently uninteresting or irrelevant backgrounds of adapting and test patterns. Our results suggest that similarity between background statistics of the adapting and test stimuli contributes to low-level visual adaptation, and that these backgrounds are thus not discarded by visual processing but provide contextual modulation of adaptation. Null cross-adaptation aftereffects must also be interpreted cautiously. These findings reduce the apparent inconsistency between psychophysical and neurophysiological data about first- and second-order stimuli.

## Highlights

• Task irrelevant backgrounds of adapting and test stimuli influence tilt aftereffect.
• Matched backgrounds enable transfer from second to first-order orientations.
• Mismatched backgrounds reduce normally large, within-order aftereffects.
• This background similarity effect is robust for weak foreground orientations.
• Null aftereffect in many studies may be partially caused by a background mismatch.

## 1 Introduction

The ubiquity of adaptation makes it a major experimental paradigm both in its own right and as a methodological tool for investigating other questions. Psychophysically, adaptation is measured by means of aftereffects, and a central issue is how the strength of such aftereffects depends on the relationship between adapting and test stimuli. It is well known that to produce strong aftereffects, adapting and test stimuli should have similar features. For example, to maximize the tilt aftereffect, the adapting and test orientations should have matched retinal location (Gibson & Radner, 1937) and spatial frequency (Ware & Mitchell, 1974). We will refer to this as the foreground similarity effect because the matched feature (e.g., spatial frequency) is a property of the foreground feature (e.g., orientation) whose adaptation is measured. The effect is easy to understand because many visual cells are jointly tuned to multiple features (e.g., orientation *and* spatial frequency), and by matching them, the adapting and test stimuli will engage maximally overlapping cell groups to produce a strong aftereffect. Indeed, the contingency of adaptation of one feature (e.g., color) on matching another feature (e.g., orientation) is viewed as evidence of joint tuning to those features (McCollough, 1965).

Using high level visual stimuli, we recently found a new form of contingent adaptation which we call the background similarity effect (Wu et al., 2009). This involves the relationship between the backgrounds rather than the foregrounds of adapting and test stimuli. For instance, adaptation to a real-face image produced a larger facial-expression aftereffect on test cartoon faces after noise with the same correlation statistics as real faces or natural images was added to the cartoon faces. This is surprising because joint tuning to facial expression and background noise is unlikely (and certainly unreported). Moreover, the background noise alone carried no facial expression and was not an integral part of, or an associated property of, the foreground faces. Thus, according to most accounts of face processing, would have been squelched or eliminated as early as possible so as not to interfere with face processing.

This study raises the question as to whether the background similarity effect for faces applies to simpler stimuli to which neurons in lower-level areas such as V1 are tuned. This is important because a great number of adaptation studies has used simple stimuli instead of faces, leading to the overwhelming consensus that second-order adaptation does not transfer to first-order stimuli (Ashida et al., 2007; Larsson, Landy, & Heeger, 2006; Nishida, Ledgeway, & Edwards, 1997; Paradiso, Shimojo, & Nakayama, 1989; Schofield, Ledgeway, & Hutchinson, 2007). The background similarity finding challenges this consensus since, by construction, first- and second-order stimuli typically have different background statistics. To our knowledge, previous studies using simple stimuli never systematically investigated the impact of this difference on the transfer of aftereffects. We therefore tested the background similarity hypothesis with the low-level, orientation tilt aftereffect. Specifically, we examined the transfer of the tilt-aftereffect from second- to first-order orientations, and also between orientations of the same type, under various manipulations of background similarity. Preliminary results were reported in an abstract (Qian and Dayan, Society for Neuroscience Abstract, 2010).

Our results demand a reevaluation of the large body of literature on cross-order adaptation, help reduce the apparent contradiction between these psychophysical studies and physiological findings on cue-invariant cells that show similar tuning to first- and second-order stimuli (Albright, 1992; Sheth et al., 1996; von der Heydt, Peterhans, & Baumgartner, 1984), and offer insights into the role of seemingly uninteresting or irrelevant backgrounds in visual processing.

## 2 Methods

### 2.1 Subjects

A total of 12 subjects consented to participate in the experiments of this study. All subjects had normal or corrected to normal vision. Experiment 1 had four subjects, Experiments 2, 3 and 4 had six subjects each. For each experiment, one subject was an author (NQ), and the rest were naive to the purpose of the study. The study was approved by the Institutional Review Board of the New York State Psychiatric Institute.

### 2.2 Apparatus

The visual stimuli were presented on a 21 in. ViewSonic (Walnut, CA) P225f monitor controlled by a Macintosh G4 computer. The vertical refresh rate was 100 Hz, and the spatial resolution was 1024 × 768 pixels. The monitor was calibrated for linearity with a Minolta LS-110 photometer. In a dimly lit room, subjects viewed the monitor from a distance of 75 cm through a black, cylindrical viewing tube (10-cm inner diameter) to exclude potential influence from external orientations. Each pixel subtended 0.029° at this distance. A chin rest was used to stabilize the head position. All experiments were run in Matlab with Psychophysics Toolbox extensions (Brainard, 1997; Pelli, 1997).

### 2.3 Visual stimuli

A round, black (0.47 cd/m^2^) fixation dot, 0.23° in diameter, was always shown at the center of the white (50.6 cd/m^2^) screen. All stimuli were grayscale in a 2.9° × 2.9° area. They included second-order, illusory lines and first-order, luminance-defined bars. We used an anti-aliasing method (Matthews et al., 2003) to ensure that the stimuli appeared smooth under the viewing condition of our experiments. In all subsequent descriptions, we define vertical orientation as 0° and orientations clockwise (CW) and counterclockwise (CCW) from vertical as positive and negative angles, respectively. The orientation of the adapting stimuli was always −15°, and the orientations of the test stimuli were within a few degrees around the vertical.

#### 2.3.1 Second-order illusory lines

**Fig. 1:**
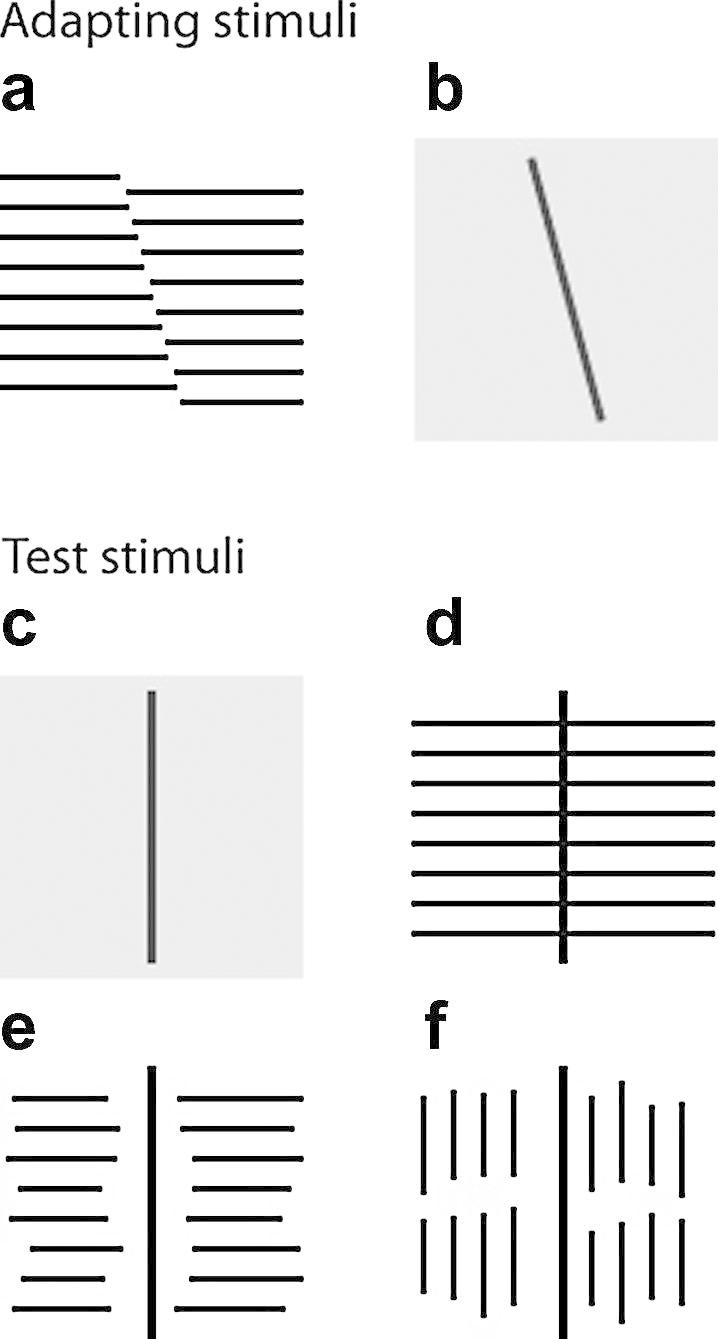
Stimuli used in Experiment 1. (a and b) The second-order and first-order adaptors with a −15° orientation (denoted as 2 and 1, respectively). (c–f) The first-order test bars on the uniform, long-horizontal-line, short-horizontal-line, and short-vertical-line backgrounds (denoted as 1, 1_H_, 1_h_, and 1_v_, respectively). Only the vertical orientation of each test set is shown here. Note that the gray levels in this and other stimuli figures are inaccurate because of format conversions, reproduction, and display dependence.

We created second-order, illusory lines by offsetting black inducing lines. In Experiment 1, a −15° illusory line was used as an adaptor (Fig. 1a); it was induced by offsetting eight evenly-spaced horizontal lines. The width of the inducing lines was 0.058° and the center-to-center vertical distance between the adjacent lines was 0.29°. In Experiment 2, illusory lines of various orientations were created by placing +45° and −45° diagonal lines on the opposite sides of the stimuli (Fig. 3). When the +45° and −45° diagonals were on the right and left sides, respectively, the resulting illusory orientations had a V-shaped background (Fig. 3, panels a and c). Conversely, when the +45° and −45° diagonals were on the left and right sides, respectively, the resulting illusory orientations had a Λ-shaped background (Fig. 3, panels b and d). The inducing lines had a width of 0.029° and the center-to-center distance in the perpendicular dimension was randomly drawn from a uniform distribution of 1–5 pixels (or 0.029° to 0.15°). A center-to-center spacing of 1 pixel means that the two adjacent lines merged into a thicker line. A −15° illusory orientation of either the V or Λ background was used as an adaptor, and a set of near-vertical illusory orientations of either the V or Λ background were used as test stimuli.

#### 2.3.2 Luminance bars

We generated first-order, luminance-defined bars of various orientations. All bars had a length of 2.6° and width of 0.087°. In Experiment 1, black, near-vertical test bars were placed on four kinds of backgrounds. The first was uniform gray (Fig. 1c) that matched the mean luminance (42.6 cd/m^2^) of the illusory adaptor (Fig. 1a). The second background was made of long horizontal lines that matched those of the inducing lines of the illusory adaptor but without the offset (Fig. 1d) and had vertical positions midway between the inducing lines of the illusory adaptor. The third background was made of short horizontal lines that did not intersect the bars (Fig. 1e). This was done by excluding the background lines from a central rectangular region of 0.46° in width. Additionally, each end of a horizontal line was reduced randomly by up to 10 pixels (0.29°) to avoid a specific illusory orientation. The fourth background was made of short vertical lines (Fig. 1f) whose lengths on average match the lengths of the short horizontal lines in the third background. These vertical background lines were also excluded from a central rectangular region of 0.46° in width but otherwise had horizontal positions that were randomized over 10 pixels (0.29°) on each side. Therefore, the distances between the test bars and the background lines did not provide reliable cues to the test bars’ orientation. For Experiment 1, we also created a −15° luminance bar on the uniform background (Fig. 1b) as an adaptor.

In Experiment 3, the black bars were placed on two kinds of background. The first was 1/*f* noise (Fig. 5, panels a and c) produced online in each trial without repetition of samples. The second was uniform gray (Fig. 5, panels b and d) that matched the mean luminance of the 1/*f* noise (25.3 cd/m^2^). The stimuli for Experiment 4 were identical to those for Experiment 3 except that the bars were gray (17.1 cd/m^2^) in order to reduce their contrast (Fig. 7). The Weber contrasts were 0.98 and 0.32 for Experiments 3 and 4, respectively.

### 2.4 Procedures

We used the method of constant stimuli for Experiment 1 and a more efficient, one-up-one-down double staircase procedure for Experiments 2–4. Subjects received no feedback on their performance at any time.

#### 2.4.1 Experiment 1

This experiment measured the tilt aftereffect transfer from the second-order, illusory orientation to the first-order, luminance orientations under various background manipulations. The main adaptor was a −15° second-order orientation stimulus (Fig. 1a, denoted as 2). The test stimuli were near-vertical first-order bars placed on four different backgrounds (Fig. 1, panels c–f, denoted as 1, 1_H_, 1_h_, and 1_v_), resulting in four adaptation conditions (denoted as 2-1, 2-1_H_, 2-1_h_, and 2-1_v_). Although these conditions had the same adaptor, we describe them as “four adaptation conditions” in order to contrast them with the corresponding no-adaptation, baseline, conditions, of which there were also four (denoted as 0-1, 0-1_H_, 0-1_h_, and 0-1_v_), one for each background. For comparison with second-order-to-first-order aftereffects, we ran a fifth adaptation condition (denoted as 1-1) to measure the first-order-to-first-order aftereffect using a −15° luminance bar on a uniform background (Fig. 1b) as the adaptor; the test luminance bars were also presented on a uniform background (Fig. 1c). Adaptation conditions 2-1 and 1-1 shared the same no-adaptation baseline condition (0-1).

The total of 9 (5 adaptation and 4 baseline) conditions were run in separate blocks, with two blocks per condition. Each test stimulus in each condition was repeated 20 times. There was a minimum 15 min break after each adaptation block to avoid carryover of any aftereffect to the next block. For the four adaptation conditions using the illusory adaptor, the block orders of pairs of conditions to be directly compared (see Section 3) were counterbalanced. The baseline conditions were always run before their corresponding adaptation condition. The trials for different test stimuli in a block were randomized. Subjects started each block of trials by fixating at the central dot and then pressing the space bar. After 500 ms, for each adaptation block the adapting stimulus appeared for 30 s in the first trial (initial adaptation) and 4 s in subsequent trials (top-up adaptation). After a 500 ms inter-stimulus interval, a test stimulus appeared for 100 ms. For the baseline blocks without adaptation, only a test stimulus was shown in each trial for 100 ms. For both adaptation and baseline trials, a 50 ms beep was then played to remind subjects to report their perception of the test stimulus. Subjects had to press the “A” or “S” key to indicate whether the perceived test orientation was CCW or CW from vertical (two-alternative forced choice). After a 1 s inter-trial interval, the next trial began.

#### 2.4.2 Experiment 2

This experiment measured the tilt aftereffects from second-order to second-order orientations under background manipulations. The adaptor was a −15° illusory line induced by either the V- and Λ-shaped background lines (Fig. 3, panels a and b, denoted as V and Λ). The test stimuli were a set of near vertical, illusory orientations, again induced by either the V- and Λ-shaped background lines (Fig. 3, panels c and d). We considered all four possible combinations of the adaptor and test backgrounds (denoted as V–V, Λ–V, Λ–Λ, and V–Λ). We also included the two no-adaptation, baseline conditions, one for each test background shape (denoted as 0–V and 0–Λ).

To speed up data collection, we used a one-up-one-down double staircase procedure for this and the following experiments. The two stairs started in opposite directions and the trials from them were randomly interleaved. Since the staircase procedure concentrated trials on the transition part of a psychometric curve, one block of 60 trials, with 30 trials per staircase, was sufficient for each condition. There was a minimum of 10 min break after each adaptation condition. All other aspects of this experiment, including counterbalancing pairs of conditions to be compared, were identical to those of Experiment 1.

#### 2.4.3 Experiment 3

This experiment measured the tilt aftereffects from first-order to first-order orientations under background manipulations. The adaptor was a −15° luminance bar on either a 1/*f* noise or uniform background (Fig. 5, panels a and b, denoted as N and U). The 1/*f* noise matches the correlation statistics of natural images (Field, 1987). The test stimuli were a set of near vertical, luminance bars, again on either a 1/*f* noise or uniform background (Fig. 5, panels c and d). We considered all four possible combinations of the adaptor and test backgrounds (denoted as N–N, U–N, U–U, and N–U). We also included the two baseline conditions for the two test backgrounds (denoted as 0–N and 0–U). All other aspects of this experiment were identical to those of Experiment 2.

#### 2.4.4 Experiment 4

Since Experiment 3 failed to show a robust background effect, we repeated it but with lower contrast adapting and test bars. We also used 4–5 more test-bar orientations to examine the psychometric functions more completely. All other aspects of this experiment were identical to those of Experiment 3.

### 2.5 Data analysis

For each condition, the test stimuli were parameterized according to their orientations, and the data were sorted to provide the fraction of clockwise responses to each test stimulus. This was done identically for the data collected with the constant-stimuli method and the double staircase method. The fractions of clockwise responses were then plotted against the parameterized test stimulus, and the resulting psychometric curve was fitted with a sigmoidal function of the form $f(x)=\frac{1}{1+e^{-a(x-b)}}$, where *a* determines the slope and *b* gives the test-stimulus parameter corresponding to the 50% point of the psychometric function [the point of subjective equality (PSE)]. An aftereffect is measured by the difference between the PSEs of the adaptation condition and the corresponding baseline condition; i.e., the horizontal shift between the midpoints of the two curves. To determine whether an aftereffect was significant, we calculated the *p* value by comparing subjects’ PSEs of the adaptation condition against those of the corresponding baseline condition via a two-tailed paired *t*-test. The same procedure was used to test whether subjects’ aftereffects or slopes under two different conditions were significantly different.

Note that the staircase procedure concentrated most trials around PSE. Consequently, some points far away from the PSE might appear noisy as only a few trials were spent on them and the subjects might accidentally press a key different from what they intended (for example, the blue circle at −5° and the red^1^ cross at 2° in Fig. 8a). This does not impact our data analysis because the sigmoid curve fit and thus the determination of the PSE were largely immune to these rare outlying points (again, see Fig. 8a).

## 3 Results

We first show that adaptation to a second-order orientation transferred more to first-order bars when the adapting and test stimuli had better matched backgrounds. We then show that the normally strong interactions among orientations of the same type could be reduced when the adapting and test stimuli had different backgrounds. We denote the vertical orientation as 0° and orientations CW and CCW from vertical as positive and negative, respectively.

### 3.1 Experiment 1: Aftereffect transfer from second-order, illusory orientation to first-order, luminance orientation

**Fig. 2:**
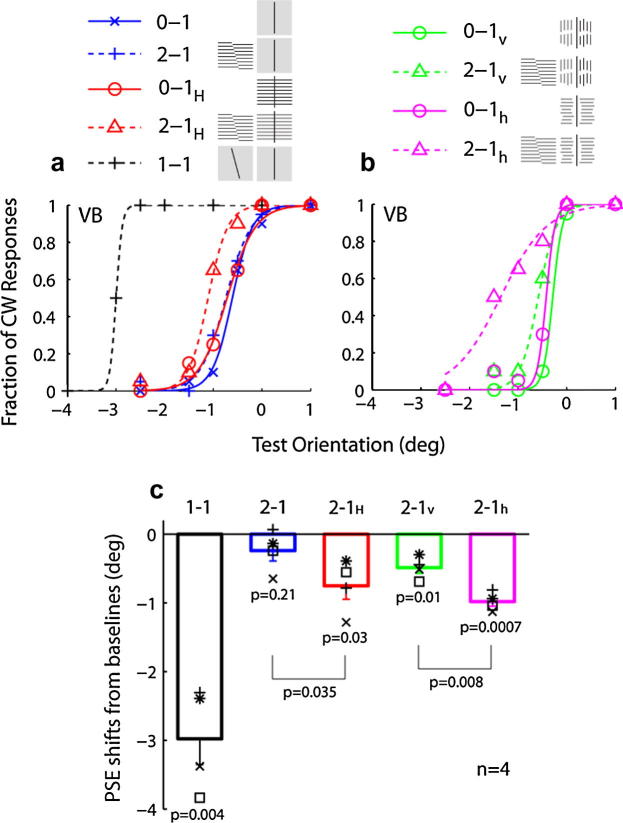
Results of Experiment 1. (a) Naïve subject VB’s psychometric curves for the 0-1, 2-1, 0-1_H_, 2-1_H_, and 1-1 conditions. (b) Subject VB’s psychometric curves for the 0-1_v_, 2-1_v_, 0-1_h_, and 2-1_h_ conditions. Each row of the legends (top) includes icons to indicate the types of adapting and test stimuli used in each condition. For the no-adaptation, baseline conditions, only the test icons are shown (first and third rows). (c) The mean tilt aftereffect of the four subjects for each adaptation condition is shown as a rectangle. The error bars represent standard errors. The symbols represent individual subjects’ aftereffects; a given symbol represents the same subject across all summary figures (Figs. 2c, 4c, 6c, and 8c). VB’s results are represented by asterisks (^*^). The *p* value for each rectangle tests whether that aftereffect is significantly different from 0. The *p* value between two rectangles tests whether the two aftereffects are significantly different from each other. Two-tailed paired *t*-tests were used.

We created a second-order, illusory contour with a −15° orientation as the adaptor (Fig. 1a), and a set of first-order, luminance bars with near-vertical orientations (the 0° vertical bar is shown in Fig. 1c) as the test stimuli. After adaptation to the second-order (abbreviated as 2) orientation, subjects judged whether the first-order (abbreviated as 1) test bars were CW or CCW from vertical. The psychometric curve for this 2-1 condition from a naïve subject is shown as blue dashed curves in Fig. 2a. We plotted the fraction of CW responses as a function of the test orientation. This curve barely shifted from the corresponding baseline condition (0-1, blue solid curves) in which the subject judged the orientation of the first-order test bars without prior adaptation (abbreviated as 0). This reproduced the well-known result that second-order adaptation does not substantially transfer to first-order stimuli (Ashida et al., 2007; Larsson, Landy, & Heeger, 2006; Nishida, Ledgeway, & Edwards, 1997; Paradiso, Shimojo, & Nakayama, 1989; Schofield, Ledgeway, & Hutchinson, 2007). For comparison, adaptation to a first-order bar, also of −15° orientation, strongly biased the perceived orientation of the first-order test bars (1-1 condition, black curve in Fig. 2a), reproducing the standard tilt aftereffect (Gibson & Radner, 1937). The leftward shift of the 1-1 condition from the 0-1 condition means that subjects perceived CW orientation more frequently after adapting to the CCW orientation.

However, if the background similarity hypothesis mentioned in the Introduction applies to low-level stimuli, then transfer from second- to first-order orientation should increase when the adapting and test stimuli have more similar backgrounds. To test this prediction, we added long horizontal lines to the test bars (Fig. 1d, abbreviated as 1_H_) to match the background of the second-order adaptor (Fig. 1a). This manipulation indeed increased the aftereffect transfer from the second- to the first-order orientations, as indicated by the curve shift of the adaptation condition (2-1_H_, red dashed curves) from the corresponding no-adaptation baseline condition (0-1_H_, red solid curves) in Fig. 2a. Since the horizontal lines added to the test stimuli were straight without offsets (Fig. 1d), this result cannot be explained by a second-order-to-second-order aftereffect.

To quantify the aftereffects and summarize the results from all four subjects, we determined the PSE – the *x*-axis point corresponding to 0.5 *y*-axis value – for each psychometric curve of each subject. We measured the aftereffect as the mean PSE shift of an adaptation condition from the corresponding baseline condition. For example, the aftereffect for the 2-1 condition is the PSE difference between the 2-1 (blue dashed) and 0-1 (blue solid) curves in Fig. 2. The four subjects’ aftereffects and their mean and SE for each adaptation condition are shown in Fig. 2c. (We represent repulsive aftereffects as negative.) The results of the subject in panels a and b are represented by asterisks (^*^). The tilt aftereffect transfer from the second- to first-order orientations was significant with matched, long-horizontal-line background (2-1_H_, red rectangle; *p* = 0.030, *t* = 3.87, df = 3), but not significant with unmatched, uniform backgrounds (2-1, blue rectangle; *p* = 0.21, *t* = 1.59, df = 3). The difference between the two aftereffects was also significant (*p* = 0.035, *t* = 3.66, df = 3). Importantly, the block order for the 2-1 and 2-1_H_ was counterbalanced across the subjects.

For reference, the black rectangle in Fig. 2c shows the mean aftereffect from the first-order-to-first-order bars on uniform background (1-1 condition). Clearly, although the background matching significantly increased the cross-class, second-order-to-first-order aftereffect transfer, the effect was small compared with the within-class, first-order-to-first-order interaction. This is not surprising because both the foreground and the background of the adapting and test stimuli were matched in the within-class case but only the backgrounds were made similar in the cross-class case.

One could argue that even though the mean luminances of the uniform and long-horizontal-line backgrounds were matched (see Section 2), other differences, instead of different degrees of similarity to the adaptor background, could be responsible for the different aftereffects between the 2-1 and 2-1_H_ conditions. For example, the intersections between the added horizontal lines and the test bars (Fig. 1d) might have biased the perceived orientation of test bars, and this bias might explain the results in Fig. 2a. This is, however, unlikely because an aftereffect was measured as a shift between an adaptation condition and its corresponding baseline condition, so any bias was subtracted if its effect was additive. The data from additional conditions described below further excluded this possibility.

If the aftereffect transfer from the second-order line to the first-order bars with the added horizontal lines was really due to the background similarity, then the transfer should become weaker if vertical lines, which do not match the adaptor background orientation, are added. To test this prediction, we generated two new sets of test stimuli by adding short horizontal (Fig. 1e, abbreviated as 1_h_) or vertical (Fig. 1f, abbreviated as 1_v_) lines to the same set of first-order test bars used in the above conditions. We used short background lines so that they did not intersect the test bars. To avoid vertical alignment of the endpoints of the background lines (which might have been subject to adaption by the illusory orientation), we randomized the endpoint positions of the background lines for each test orientation. The total lengths of the background lines were the same, on average, for the two backgrounds; this ensures that the mean background luminances, and thus the effective contrasts of the test bars, were the same for the backgrounds. The distances between the test bars and the nearest vertical background lines on either side were separately randomized so that they did not provide reliable cues for the orientations of the test bars (see Section 2).

We then measured the transfer of the tilt aftereffect from the second-order adaptor (Fig. 1a) to these test bars shown with the two different background orientations. The psychometric curves from the same naïve subject are shown in Fig. 2b. The magenta dashed and solid curves are the psychometric functions for the adaptation (2-1_h_) and baseline (0-1_h_) conditions when the test bars had the short-*horizontal*-line background. The green dashed (2-1_v_) and solid (0-1_v_) curves are the corresponding results when the test bars had the short-*vertical*-line background. The shifts between the psychometric curves of the same color indicate that, as predicted, the test bars with the horizontal background produced a larger aftereffect than those with the vertical background. The mean aftereffects from the same four subjects are summarized as the magenta and green rectangles in Fig. 2c for the horizontal and vertical backgrounds, respectively, with a significant difference between them (*p* = 0.0081, *t* = 6.30, df = 3; the block order for these two conditions was counterbalanced). Thus, the aftereffect transfer from second-order to first-order orientations depends on the similarity of the background orientations between the adapting and test stimuli.

Interestingly, although the aftereffect transfer for the vertical background was smaller than that for the horizontal background, it was still significant (green rectangle in Fig. 2c; *p* = 0.0094, *t* = 5.97, df = 3), and was larger than that for the uniform background (blue rectangle in Fig. 2c) though not significantly (*p* = 0.20, *t* = 1.66, df = 3). This is perhaps because, like the adaptor, the vertical background did have lines (albeit of the wrong orientation), whereas the uniform background did not contain any line at all.

One might argue that the vertical background reduced the saliency of the near-vertical test bars more than the horizontal background did, because of the stronger crowding effect or attentional distraction among more similar items (Levi, 2008) or texture suppression (Knierim & van Essen, 1992; Li, 2000). This is unlikely because the test bars were thicker and much longer than the background lines and so they stood out. To exclude this possibility formally, we measured the slopes of the psychometric curves and tested their dependence on background orientation. If the test bars were less salient on the vertical background, then the slopes, indicating orientation discriminability, would be shallower for this background. We found that the slopes varied widely and the mean slope (averaged over the adaptation and baseline conditions of the four subjects) was 0.21/deg for the vertical background and 0.15/deg for the horizontal background; the difference, which was in any case, in the opposite direction of the saliency prediction, was not significant (*p* = 0.31, *t* = 1.10, df = 7). This suggests that saliency did not play a part in our results.

We finally note that across all summary figures of this paper (Figs. 2c, 4c, 6c, and 8c), 12 different symbols are used consistently to represent the aftereffects of the twelve subjects. The plus (+) symbol represents an author (NQ)’s data; all other symbols represent data from naïve subjects.

### 3.2 Experiment 2: Aftereffect from second-order to second-order orientations

In Experiment 1, we focused on the transfer of the aftereffect from second- to first-order orientations. By construction, stimuli of different orders typically have very different backgrounds. We showed that we could significantly increase the aftereffect by properly matching the backgrounds of the adapting and test stimuli. In this and subsequent experiments, we considered the converse question as to whether the normally strong adaptation interactions among the stimuli of the same type can be reduced by deliberately introducing different backgrounds to the adapting and test stimuli.

**Fig. 3:**
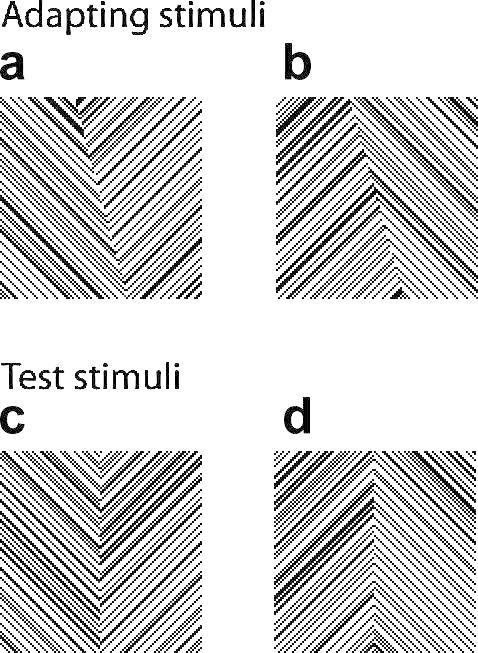
Stimuli used in Experiment 2. (a and b) The second-order adaptors with a −15° orientation induced by the V- and Λ-shaped background lines (denoted as V and Λ, respectively). (c and d) The second-order test stimuli induced by the V- and Λ-shaped background lines, respectively. Only the vertical orientation of each test set is shown here.

In Experiment 2, we measured the tilt aftereffect from adaption between the same type of second-order stimuli under background manipulations. We generated second-order, illusory orientations using inducing lines that formed either a V- or Λ-shaped background. The adaptor was a −15° illusory line with either background shape (Fig. 3a and b). The test stimuli were a set of near vertical, second-order lines, again with either background shape (Fig. 3c and d). We considered all four possible combinations of the adaptor and test backgrounds; they are denoted as V–V, Λ–V, Λ–Λ, and V–Λ conditions, where, for example, Λ–V means that the adaptor had a Λ background and the test set all had a V background. We also included the two baseline conditions without adaptation using the test stimuli with the two backgrounds, and they are denoted 0–Λ and 0–V conditions. The order of the V–V and Λ–V conditions, and that of the Λ–Λ, and V–Λ conditions were counterbalanced across the subjects. Moreover, if a subject ran the V–V condition *after* the Λ–V condition, then he/she ran the Λ–Λ condition *before* the V–Λ condition.

**Fig. 4:**
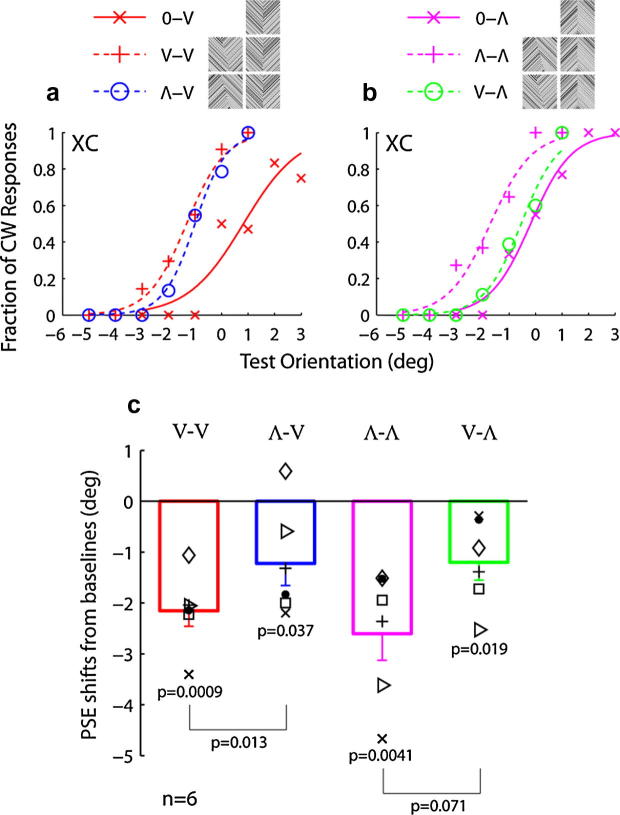
Results of Experiment 2. (a) Naïve subject XC’s psychometric curves for the 0–V, V–V, and Λ–V conditions. (b) Subject XC’s psychometric curves for the 0–Λ, Λ–Λ, and V–Λ conditions. Each row of the legends (top) includes icons to indicate the types of adapting and test stimuli used in each condition. For the no-adaptation, baseline conditions, only the test icons are shown (first rows). (c) The mean tilt aftereffect of the six subjects for each adaptation condition is shown as a rectangle. The error bars represent standard errors. The symbols represent individual subjects’ aftereffects; a given symbol represents the same subject across all summary figures (Figs. 2c, 4c, 6c, and 8c). XC’s results are represented by filled dots. The *p* value for each rectangle tests whether that aftereffect is significantly different from 0. The *p* value between two rectangles tests whether the two aftereffects are significantly different from each other. Two-tailed paired *t*-tests were used.

The psychometric curves from a naïve subject are shown in Fig. 4, panels a and b. The 0–V, V–V, and Λ–V conditions are in panel a as red solid, red dashed, and blue dashed curves, and the 0–Λ, Λ–Λ, and V–Λ conditions are in panel b as magenta solid, magenta dashed, and green dashed curves. The V–V curve shifted more than the Λ–V curve, and the Λ–Λ curve shifted more than the V–Λ curve, from the corresponding baseline conditions, 0–V and 0–Λ, respectively, indicating that the second-order-to-second-order aftereffects were larger when the adaptor and test stimuli had more similar backgrounds. It is interesting to note that, for this subject, the background mismatch reduced the V–Λ aftereffect more than the Λ–V aftereffect; other subjects showed the opposite behavior (see Fig. 4c).

The six subjects’ aftereffects and their mean and SE for each adaptation condition are summarized in Fig. 4c. The results of the subject in panels a and b are represented by filled dots. The difference between the V–V and Λ–V aftereffects was significant (*p* = 0.013, *t* = 3.80, df = 5). The difference between the Λ–Λ and V–Λ aftereffects, however, failed to reach significance (*p* = 0.071, *t* = 2.29, df = 5). This is mainly due to one subject, represented by crosses (x), who had a very large Λ–Λ aftereffect but a small V–Λ aftereffect. Paradoxically, although his data were highly consistent with our background similarity hypothesis, they increased the inter-subject variability in the difference between the Λ–Λ and V–Λ aftereffects, rendering the difference non-significant. If this subject’s data were excluded, then the difference between the Λ–Λ and V–Λ aftereffects became significant (*p* = 0.010, *t* = 4.58, df = 4), and the difference between the V–V and Λ–V aftereffects remained significant (*p* = 0.040, *t* = 3.00, df = 4).

Since our main goal was to test the background similarity hypothesis, we pooled the same-background conditions (V–V and Λ–Λ) and pooled the orthogonal background conditions (Λ–V and V–Λ) without excluding any subject, and found that the difference between the two pooled data sets was highly significant (*p* = 0.0040, *t* = 3.62, df = 11).

### 3.3 Experiment 3: Aftereffect from first-order to first-order orientations

**Fig. 5:**
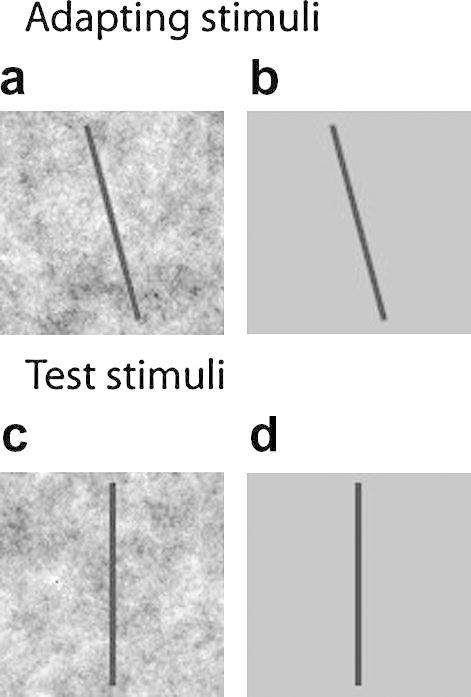
Stimuli used in Experiment 3. (a and b) The first-order adaptors with a −15° orientation on a 1/*f* noise and a uniform background (denoted as N and U, respectively). (c and d) The first-order test stimuli on a 1/*f* noise and a uniform background, respectively. Only the vertical orientation of each test set is shown here.

In this experiment, we examined whether the normally strong tilt aftereffect from adaptation between the first-order orientations could be reduced by deliberately introducing different backgrounds underneath the adapting and test stimuli. We generated first-order, luminance bars on either a 1/*f* noise (N) or a uniform (U) background. The mean luminance of these two types of backgrounds was matched. The adaptor was a −15° bar on either background (Fig. 5a and b). The test stimuli were a set of near-vertical bars, again on either background (Fig. 5c and d). We considered all four possible combinations of the adaptor and test backgrounds; they are denoted as N–N, U–N, U–U, and N–U conditions, where, for example, U–N means that the adaptor was on the uniform background and the test bars were all on the 1/*f* noise background. We also included the two baseline conditions without adaptation using the test bars on the two backgrounds, and they are denoted as 0–N and 0–U conditions. A new noise sample was generated online for each instance without repetition of a specific noise pattern. The counterbalancing of the order of different conditions was identical to that of Experiment 2.

**Fig. 6:**
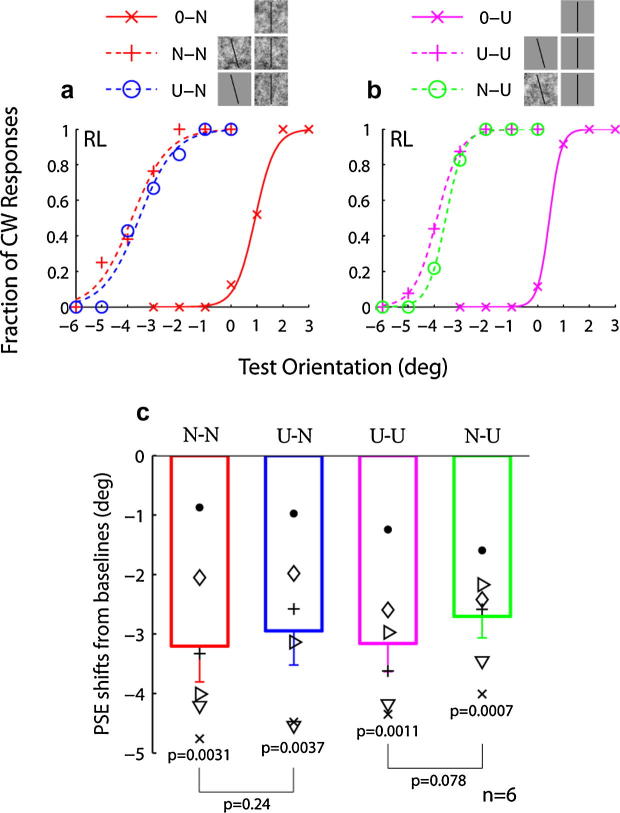
Results of Experiment 3. (a) Naïve subject RL’s psychometric curves for the 0–N, N–N, and U–N conditions. (b) Subject RL’s psychometric curves for the 0–U, U–U, and N–U conditions. Each row of the legends (top) includes icons to indicate the types of adapting and test stimuli used in each condition. For the no-adaptation, baseline conditions, only the test icons are shown (first rows). (c) The mean tilt aftereffect of the six subjects for each adaptation condition is shown as a rectangle. The error bars represent standard errors. The symbols represent individual subjects’ aftereffects; a given symbol represents the same subject across all summary figures (Figs. 2c, 4c, 6c, and 8c). RL’s results are represented by crosses (x). The *p* value for each rectangle tests whether that aftereffect is significantly different from 0. The *p* value between two rectangles tests whether the two aftereffects are significantly different from each other. Two-tailed paired *t*-tests were used.

The psychometric curves from a naïve subject are shown in Fig. 6, panels a and b. The 0–N, N–N, and U–N conditions are in panel a as red solid, red dashed, and blue dashed curves, and the 0–U, U–U, and N–U conditions are in panel b as magenta solid, magenta dashed, and green dashed curves. The N–N curve shifted slightly more than the U–N curve, and the U–U curve shifted slightly more than the N–U curve, from the corresponding baseline conditions, 0–N and 0–U, respectively. The six subjects’ aftereffects and their mean and SE for each adaptation condition are summarized in Fig. 6c. The results of the subject in panels a and b are represented by crosses (x). The difference between the N–N and U–N aftereffects (*p* = 0.24, *t* = 1.32, df = 5), and that between U–U and N–U aftereffects (*p* = 0.078, *t* = 2.20, df = 5), were very small and not significant. However, the difference between the pooled same-background conditions (N–N and U–U) and the pooled different-background conditions (U–N and N–U) was significant (*p* = 0.026, *t* = 2.57, df = 11). We conclude that for the first-order bars used in this experiment, the background similarity effect was either absent or weak, compared with that for the second-order stimuli in Experiment 2.

The two subjects represented by crosses (x) and pluses (+) showed larger aftereffects for the U–U condition in this experiment than those for the 1-1 condition in Experiment 1 even though the two conditions were quite similar. One possibility is that the constant-stimuli method for Experiment 1 underestimated the aftereffect (Geesaman & Qian, 1998) because the range of test orientations for the 1-1 condition did not symmetrically bracket the PSEs in the middle; this made the subjects’ CW responses far out-numbered the CCW responses and the subjects tended to balance the two responses a little, reducing the aftereffect. The double-staircase procedure for this experiment did not have the same problem because a broader range of test orientations were used and more importantly, the procedure quickly zoomed into the region around PSE where the CW and CCW responses were equally likely.

### 3.4 Experiment 4: Aftereffect from first-order to first-order orientations under reduced contrast

**Fig. 7:**
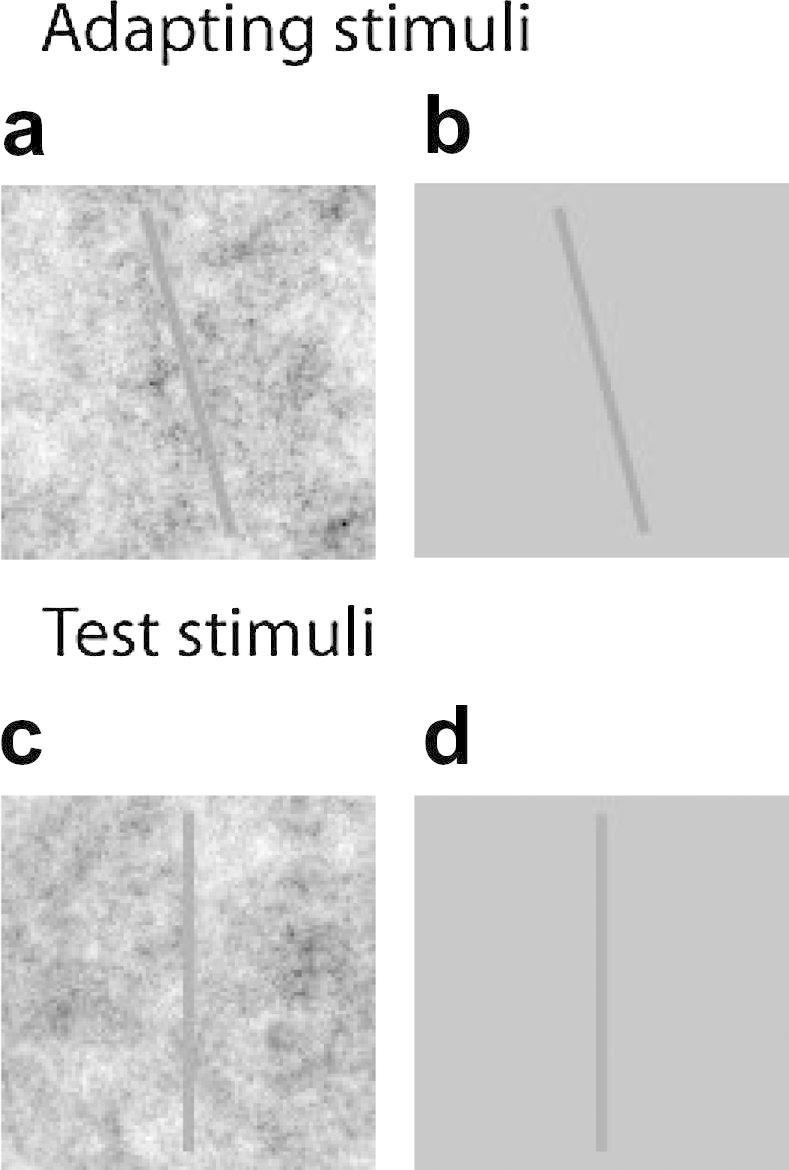
Stimuli used in Experiment 4. The bar contrast was reduced compared with that for Experiment 3. (a and b) The first-order adaptors with a −15° orientation on a 1/*f* noise and a uniform background (denoted as N and U, respectively). (c and d) The first-order test stimuli on a 1/*f* noise and a uniform background, respectively. Only the vertical orientation of each test set is shown here.

One possible explanation for the relatively weak effect in Experiment 3 is that the bars had such high contrast that the *foreground* similarity effect overwhelmed any background manipulation. This explanation would also be consistent with the large background similarity effect for the second-order stimuli in Experiment 2, since second-order stimuli are generally not as salient as the first-order ones. We tested this explanation in Experiment 4 by reducing the contrast of the test bars (Fig. 7), but otherwise running the same conditions as in Experiment 3.

**Fig. 8:**
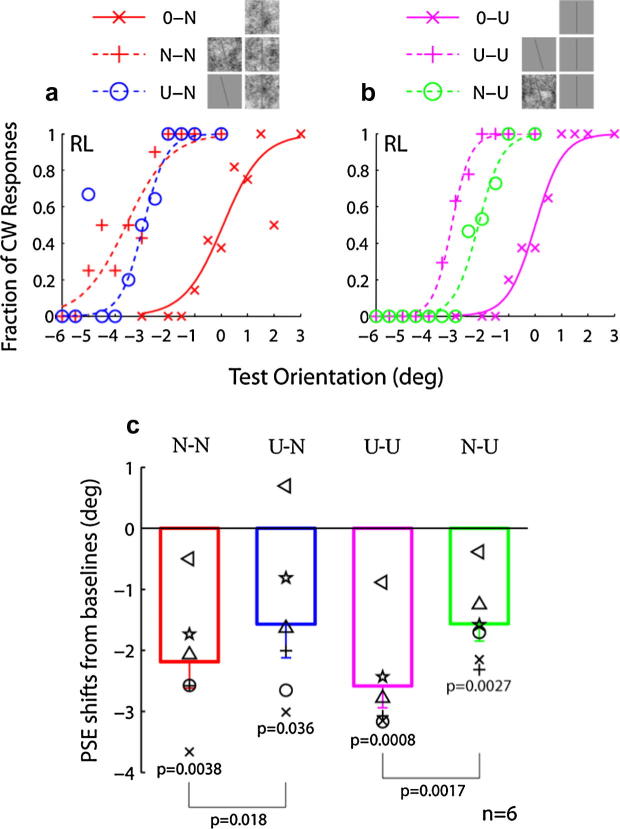
Results of Experiment 4. (a) Naïve subject RL’s psychometric curves for the 0–N, N–N, and U–N conditions. (b) Subject RL’s psychometric curves for the 0–U, U–U, and N–U conditions. Each row of the legends (top) includes icons to indicate the types of adapting and test stimuli used in each condition. For the no-adaptation, baseline conditions, only the test icons are shown (first rows). (c) The mean tilt aftereffect of the 6 subjects for each adaptation condition is shown as a rectangle. The error bars represent standard errors. The symbols represent individual subjects’ aftereffects; a given symbol represents the same subject across all summary figures (Figs. 2c, 4c, 6c, and 8c). The *p* value for each rectangle tests whether that aftereffect is significantly different from 0. The *p* value between two rectangles tests whether the two aftereffects are significantly different from each other. Two-tailed paired *t*-tests were used.

The psychometric curves from a naïve subject are shown in Fig. 8, panels a and b; for comparison, we picked the same subject whose data were shown in Fig. 6, panels a and b, for Experiment 3. The 0–N, N–N, and U–N conditions are shown in panel a as red solid, red dashed, and blue dashed curves, and the 0–U, U–U, and N–U conditions are shown in panel b as magenta solid, magenta dashed, and green dashed curves. Compared with Fig. 6, the differences between the N–N and U–N conditions, and between the U–U and N–U conditions were more pronounced. The six subjects’ aftereffects and their mean and SE for each adaptation condition are summarized in Fig. 8c. The difference between the N–N and U–N aftereffects (*p* = 0.018, *t* = 3.46, df = 5), and that between U–U and N–U aftereffects (*p* = 0.0017, *t* = 6.13, df = 5), were both significant. The difference between the pooled same-background conditions (N–N and U–U) and the pooled different-background conditions (U–N and N–U) was highly significant (*p* = 0.000064, *t* = 6.24, df = 11). We conclude that reducing the contrast of the first-order bars makes the background similarity effect larger and more robust.

Although the 1/*f* noise and uniform backgrounds had the same mean luminance, it appeared that the former rendered the foreground bars less salient than did the latter. We confirmed this by comparing the psychometric slopes between the conditions with the test bars on the 1/*f* noise background (0–N, N–N, and U–N) and the conditions with the test bars on the uniform background (0–U, U–U, and N–U). The mean slopes were 0.47 and 0.63/deg, respectively, which are very significantly different (*p* = 0.0019, *t* = 3.66, df = 17). However, it is important to note that this saliency difference cannot explain the pattern of results in Fig. 8. Based solely on saliency, one would expect aftereffects to be larger when the adapting stimulus is more salient, and the test stimuli are less salient. Therefore, since the bar is more salient on the uniform, than the 1/*f* noise background, we would expect the U–N condition to produce the largest aftereffect, the N–U condition to produce the smallest aftereffect, and the N–N and U–U conditions to produce intermediate aftereffects. However, Fig. 8 shows that the aftereffects of the N–U and U–N conditions were not significantly different from each other (*p* = 0.99, *t* = 0.019, df = 5), and they were significantly smaller than those of the N–N and U–U conditions (all *p*’s < 0.039, *t*’s > 2.78, df’s = 5 for the four comparisons). We therefore conclude that background similarity, rather than saliency, explains the results in Fig. 8.

## 4 Discussion

In this study, we demonstrated that simple oriented stimuli, to which tuning starts as early as V1 (Hubel & Wiesel, 1968), exhibit a significant background similarity effect. We first reproduced the well-known finding that adaptation to second-order orientation does not transfer well to first-order orientation. We then showed that the transfer increased significantly when the backgrounds of the adapting and test stimuli were better matched. We further showed that when the background orientations of the second-order adaptor and first-order test stimuli changed from being the same to being orthogonal, the aftereffect transfer decreased. Finally, we showed that the normally strong adaptation among orientations of the same type could often, though not always, be reduced when the adapting and test stimuli had different backgrounds. This reduction consistently occurred when the foreground orientations were relatively weakly salient, presumably because the foreground similarity effect did not overwhelm the background similarity effect. However, salience, by itself, could not explain our results; rather it appeared to modulate the background effect.

Just as for face stimuli (Wu et al., 2009), the background similarity effect for oriented stimuli depends on both first- and higher-order image statistics. For example, in Experiment 1, the test backgrounds with the short horizontal and vertical lines had the same first-order luminance distribution. The horizontal background better matched the higher-order statistics of the adapting illusory orientation, and produced a larger transfer of the aftereffect.

### 4.1 Alternative explanations

Our experiments explicitly ruled out various alternative explanations of our data, including intersections between background lines and test bars (Experiment 1) and differential saliency of test bars on different backgrounds (Experiments 1 and 4). One additional factor has been suggested that is also important to consider, namely that first-order-to-first-order adaptation could have affected the processing of the backgrounds of the test stimuli, thereby changing the way their foregrounds were perceived. For example, in Experiment 1, we showed that adding horizontal lines to the test bars increased the tilt aftereffect transfer from the illusory-line adaptor to the test bars. However, one might argue that this increased transfer was attributable not to better matched backgrounds but to first-order-to-first-order adaptation between the horizontal inducing lines of the adaptor and the horizontal background lines of the test bars. Specifically, the offset and length gradient of the inducing lines could have introduced an asymmetry in this first-order-to-first-order adaptation and thus have led to the observed result. We believe that this is unlikely, because there is no tilt aftereffect on horizontal (test) lines from horizontal (adapting) lines, and, in any case, the task was to judge the orientation of near-vertical test bars, not the orientation of horizontal background lines. Likewise, in Experiment 2, the first-order-to-first-order adaptation was between the diagonal inducing lines of either the same or orthogonal orientations and must produce no aftereffect, and the task was to judge the orientation of the near-vertical illusory lines, not the diagonal inducing lines. Moreover, stimuli in Experiment 4 did not contain background lines or length gradients, and thus the result could not be explained by an asymmetric first-order-to-first-order adaptation. Taken together, we suggest that our experiments are more parsimoniously explained by the background similarity hypothesis than by the alternatives.

### 4.2 Functional interpretations and neural mechanisms of the background similarity effect

It is commonly assumed that to transmit and process information efficiently, the visual system should extract the relevant features of input stimuli and discard the irrelevant background as quickly as possible. For instance, Fig. 7a shows a luminance bar on a 1/*f* noise background; one might expect the noise to be swiftly eliminated, since it can only corrupt the estimation of the (task-relevant) orientation of the bar. However, our study suggests that this expectation is not entirely fulfilled, as the seemingly uninteresting or irrelevant background can significantly influence adaptation aftereffects (at least when the bars have suitably low contrast). We found that it is not necessary to replicate the exact background pattern, since we only matched the statistics, and not the pixels, of the noise in the N–N condition (and since vertical background bars partially restored second-order to first-order transfer in Experiment 1). However, the precise statistics that characterize the similarity of the background textures are not clear.

Adaptation aftereffects concern temporal interactions between stimuli. Thus, the background similarity effect could be a mechanism allowing the statistical dissimilarity of stimuli to limit over-generalization of their temporal interactions. It has been shown that adaptation to one face type (e.g., monkey) often has a greatly reduced impact on subsequent perception of another type (e.g., human), compared with strong interactions within the same face category (Fox & Barton, 2007; Little et al., 2008; Ng et al., 2006; Rhodes et al., 2004; Wu et al., 2009; Yamashita et al., 2005). The background similarity effect may be a contributing factor to such category contingent aftereffects, since different types of faces likely have different background, as well as foreground, statistics.

As mentioned in the Introduction, classic contingent aftereffects such as McCollough effect (McCollough, 1965) or spatial-frequency-contingent tilt aftereffect (Ware & Mitchell, 1974) may be viewed as involving a foreground similarity effect; they can be explained by, and are taken as evidence for, joint coding of the foreground features involved such as color and orientation, or spatial frequency and orientation. By the same reasoning, the background similarity effect then predicts joint coding of foreground features and background statistics. However, visual cells are not known to be particularly responsive, let alone selective, to featureless noise backgrounds like those in Fig. 7. Rather than being coded, as in the traditional sense of tuning curves, the background statistics might modulate the tuning of cells to stimulus features. Adaptation could affect, and aftereffects could depend on both the tuning of the foreground features, and modulation associated with the background statistics. Physiological studies would be required to resolve this issue.

### 4.3 First- and second-order stimuli and cue invariance

As also mentioned in the Introduction, there is a large body of literature on cross-order adaptation using low-level stimuli such as orientation and motion (Ashida et al., 2007; Larsson, Landy, & Heeger, 2006; Nishida, Ledgeway, & Edwards, 1997; Paradiso, Shimojo, & Nakayama, 1989; Schofield, Ledgeway, & Hutchinson, 2007). The overwhelming consensus has been that second-order adaptation does not transfer to first-order stimuli. This has duly been interpreted as indicating that their processing is separate. However, this interpretation has two problems. First is a directional asymmetry: first-order adaptation does often, though not always, transfer to second-order stimuli. A common explanation is that there are first-order cues in the second-order stimuli so that the transfer is really first-order to first-order; however, it is then not clear why transfer would not then occur when the second-order stimuli are the adaptors instead. The second problem is that cue-invariant cells with similar tuning to first- and second-order stimuli have been found in many visual areas, including low-level areas such as V1, V2, and MT (Albright, 1992; Sheth et al., 1996; von der Heydt, Peterhans, & Baumgartner, 1984). It becomes puzzling why these cells would not form the basis of a robust transfer of the aftereffect from second- to first-order stimuli.

Our results help reduce these problems by showing that second- to first-order transfer does occur at a psychophsyical level, provided that the backgrounds of the adapting and test stimuli are well matched. Note that previous physiological studies did not use similar backgrounds for first- and second-order stimuli, and revealed varying degrees of separate and shared processing of first- and second-order stimuli in different cells. This variation could arise from differences in the aspects of the backgrounds to which they are sensitive, with some being at least somewhat cue invariant without our background manipulations, and others requiring the background to be more evidently similar. We thus predict that more similar background statistics increase either the fraction of cue-invariant cells or the degree of cue invariance of the same fraction of cells. It would be interesting to test this prediction physiologically, as confirmation would uncover a novel non-classical influence on visual responses.

In summary, we have demonstrated that the background similarity effect is a general phenomenon in visual adaptation that applies to both simple and complex stimuli. Functionally, it suggests the visual system uses the background statistics of stimuli to gate their temporal interactions so as to reduce over-generalization of aftereffects. Psychophysically, it calls for a reexamination of a large body of literature on null aftereffect transfer from second- to first-order stimuli, and reduces the disagreement with the physiological finding of cue-invariant cells. Physiologically, we speculate that the background statistics may modulate the tuning of foreground features and the degree of the cue invariance of visual cells. Further studies will be needed to establish the neural mechanisms of the background similarity effect, and to provide a quantitative measure of similarity.

## Footnotes

1 For interpretation of color in Figs. 2, 4, 6 and 8, the reader is referred to the web version of this article.
